# Adult Anomalous Left Coronary Artery Arising From the Pulmonary Artery (ALCAPA) Syndrome as First Presentation With Atrial Fibrillation in a Marathon Runner

**DOI:** 10.7759/cureus.15354

**Published:** 2021-05-31

**Authors:** Mrhaf Alsamman, Kubra Tuna, Sandi Dunn, Faysal Rifai, Justin Reed

**Affiliations:** 1 Internal Medicine, Health Corporation of America - University of Central Florida (HCA-UCF) Consortium, Ocala Regional Medical Center, Ocala, USA; 2 Internal Medicine, Health Corporation of America (HCA) Ocala Regional Medical Center, Ocala, USA

**Keywords:** anomalous origin of the left coronary artery from pulmonary artery (alcapa), mitral valve insufficiency, congenital cardiac anomalies, stroke, atrial fibrillation

## Abstract

Anomalous left coronary artery arising from the pulmonary artery (ALCAPA) syndrome is a very rare congenital heart disease with an incidence of one in 300,000 and a high rate of mortality early in life if left untreated. Adult-type ALCAPA presents when significant collaterals develop from the right coronary artery (RCA) to the left coronary artery (LCA). Even with the collaterals, chronic sub-endocardial ischemia occurs in most cases, and patients die from sudden cardiac death.

Here we present a case of a 38-year-old female who lived an active and healthy life and presented with chest pain and palpitations. Initial electrocardiography (EKG) showed atrial fibrillation with rapid ventricular response. Although initial cardiac enzymes were negative and there were no ischemic EKG changes, troponins became elevated over the course of the hospital stay and the patient underwent a left heart catheterization. Results revealed a dilated RCA extending to the left ventricle and an anomalous left main originating from the pulmonary artery with complete occlusion. The patient received medical management for acute coronary syndrome, including heparin infusion for 48 hours, aspirin, metoprolol, and atorvastatin. She was referred to a tertiary care facility for surgical correction of anomaly of the coronary arteries. The next day, the patient arrived in the emergency department with an acute onset of speech difficulty and left-sided weakness. A brain CT without contrast showed hematoma in the right frontal lobe. The patient underwent surgical evacuation of the hematoma with marked improvement of her weakness. The patient recovered after a successful surgical repair involving translocation of the left main coronary artery to the aorta.

It has been reported that ALCAPA should be considered in a young adult with dilated cardiomyopathy and mitral regurgitation (MR). Other common presentations include acute myocardial infarction, angina, and dyspnea on exertion. Sudden cardiac death is not uncommon; however, it tends to decrease with age of diagnosis. Interestingly, our patient was known to have MR with regular follow-up at the cardiology clinic for years. Echocardiogram never showed any abnormalities other than MR. She never received further workup to address the reason of MR, although she has no underlying chronic conditions that can explain it. In relatively young patients with a healthy lifestyle presenting with chest pain, a broader look at etiologies should be considered. We would like to emphasize the importance of looking up for possible coronary artery disease, especially in young individuals.

## Introduction

Anomalous left coronary artery arising from the pulmonary artery (ALCAPA) is also known as Bland-White-Garland syndrome. The first case of ALCAPA syndrome was published in 1993 by Bland, White, and Garland. This is a very rare congenital heart disease with a high mortality rate near 90% in the first year of life if not treated. Fewer patients survive beyond the first year of life if adequate collateral circulation is present [1]. Congenital coronary abnormalities are present in approximately 0.3-0.8% of the population. ALCAPA syndrome is estimated to comprise 0.24-0.46% of all congenital heart disease cases [2]. Due to the presence of a patent ductus arteriosus and physiologic pulmonary hypertension, this disease is challenging to diagnose in the first months of life. Similarly, diagnosis in a living adult is rare, as most information involving ALCAPA syndrome is from case reports and patients who have been resuscitated and survived [2]. On the contrary, our patient presented with chest pain and palpitations secondary to atrial fibrillation, as observed on admission. The patient also developed a stroke, which resulted in left-sided weakness, two days after initial presentation.

## Case presentation

A 38-year-old female with a past medical history of mitral regurgitation (MR) presented with a sudden onset of chest pain, palpitations, and diaphoresis during exercise, which did not resolve with rest. The patient was a marathon runner and normally in good health. The only medical problem that she was aware of was MR, which was found on a routine health examination at the age of 24 years. For this, she followed up with a cardiologist and received routine echocardiograms every two years.

On presentation, the patient’s vitals revealed an irregular heart rate in the 150s and a blood pressure of 88/47 mmHg. Atrial fibrillation with a rapid ventricular response (RVR) was observed on the electrocardiogram, in which her heart rate did not respond to bolus intravenous (IV) fluid resuscitation, metoprolol, or a loading dose of amiodarone. The cardiology service was consulted, which recommended to continue with fluid resuscitation and hold further heart rate control agents due to low blood pressure. On laboratory workup, hypokalemia was found and potassium replacement was given. Otherwise, magnesium and thyroid-stimulating hormone (TSH) were within normal limits (Table 1).

**Table 1:** Patient’s initial serum biochemistry results. BUN, blood urea nitrogen; BNP, brain natriuretic peptide; TSH, thyroid-stimulating hormone

Investigation	Value	Normal Value
Sodium	135 mmol/L	136-145 mmol/L
Potassium	3.2 mmol/L	3.5-5.1 mmol/L
Chloride	102 mmol/L	98-107 mmol/L
Bicarbonate	17 mmol/L	22-34 mmol/L
BUN	10 mg/dL	7-18 mg/dL
Creatinine	1.1 mg/dL	0.6-1.3 mg/dL
Glucose	249 mg/dL	70-110 mg/dL
Troponin	<0.012 ng/mL	0.00-0.034 ng/mL
Magnesium	2.2 mg/dL	1.8-2.5 mg/dL
BNP	447 pg/mL	0.0-450 pg/mL
TSH	2.88 uIU/mL	0.34-5.6 uIU/mL
Hemoglobin A1C	5.0 %	4.8-6.1 %

**Figure 1:**
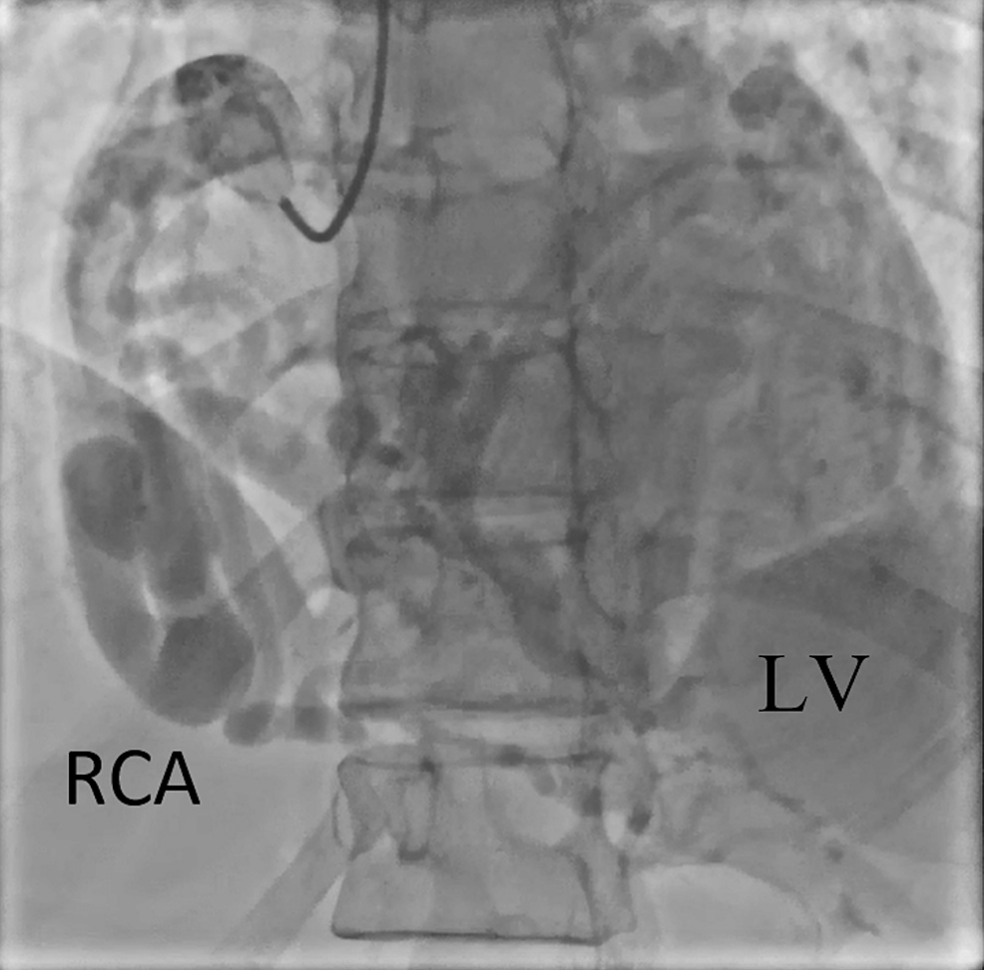
Coronary angiography showing RCA dilation and tortuosity, with right circulation extending to the LV. RCA, right coronary artery; LV, left ventricle

**Figure 2:**
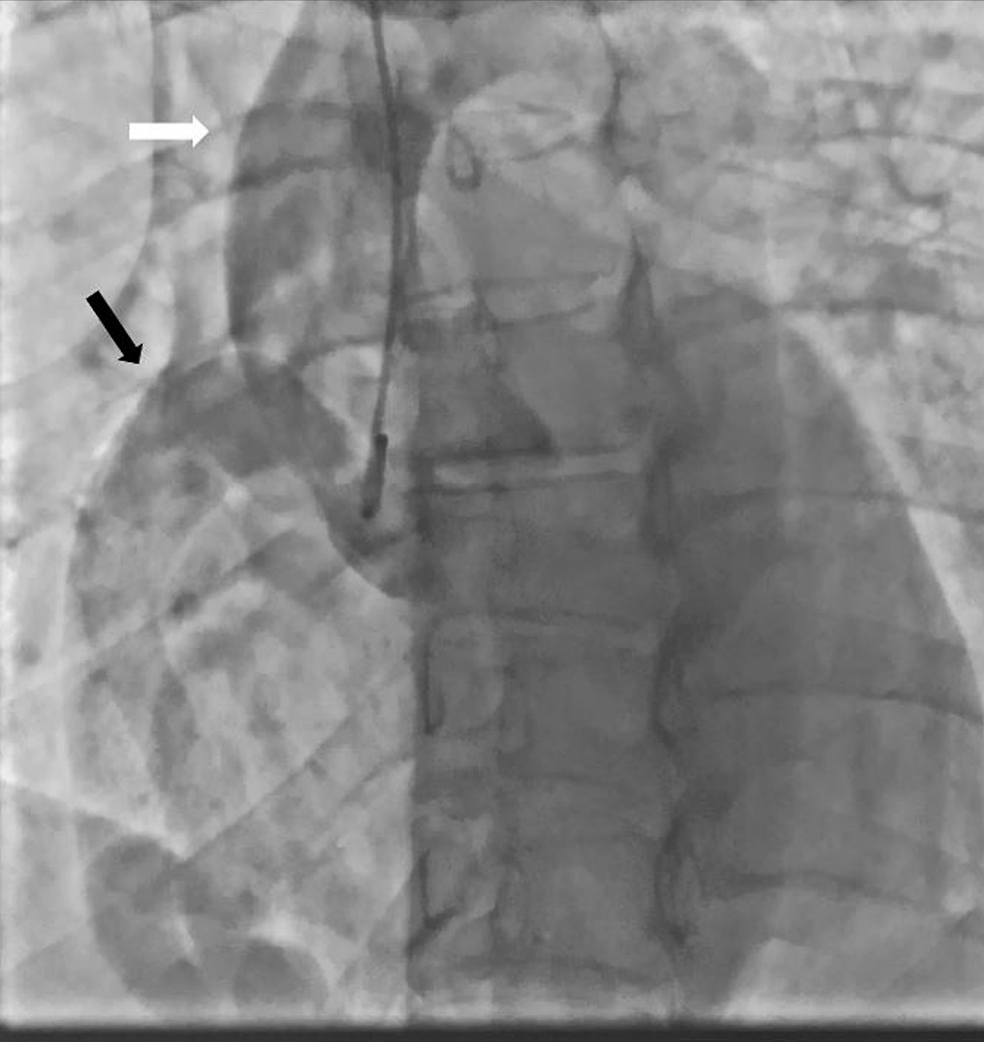
Coronary angiography showing the size of the right coronary artery (black arrow) compared to the size of the ascending aorta (white arrow).

**Figure 3:**
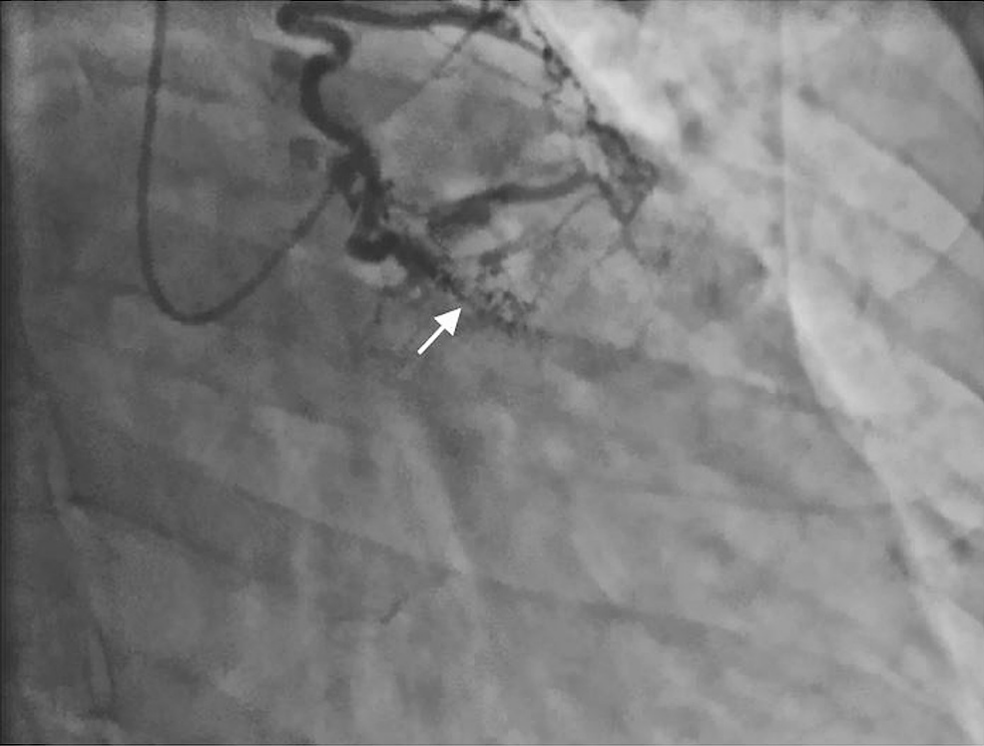
Coronary angiography demonstrating the left coronary circulation. The left descending artery is occluded (white arrow).

Initial troponin was negative, but six hours later it increased to 15.8 ng/mL and further increased to 18.5 ng/mL on a subsequent measurement. IV heparin therapy was initiated, and the patient underwent urgent cardiac catheterization. The cardiac angiogram showed a markedly ectatic right coronary artery (RCA) (Figures 1, 2). The left main coronary artery (LMCA) appeared to be anomalous. The left descending artery (LAD) and left circumflex artery (LCx) were not visualized in their usual position due to a possibly occluded LAD. There appeared to be a fistula involving a branch of the LAD (Figure 3). Echocardiogram revealed an ejection fraction (EF) of 55%, moderate MR, and mildly dilated left atrium, and right ventricle systolic pressure was estimated to be 52 mmHg.

There was no intervention performed during the cardiac catheterization due to the anomalous coronary vessels. Coronary computed tomography angiogram (CCTA) was planned in order to get a better idea of the anatomy of the coronaries, and the cardiothoracic surgery service was consulted. The patient spontaneously converted to sinus rhythm, with improvement in her heart rate and blood pressure. The local cardiothoracic surgeon recommended to transfer the patient to a tertiary care facility for further evaluation by a congenital heart specialist. Further workup including CCTA was scheduled, and the guideline-recommended therapy was initiated with aspirin, metoprolol, and atorvastatin. The patient was discharged with an outpatient appointment at the tertiary care facility.

**Figure 4:**
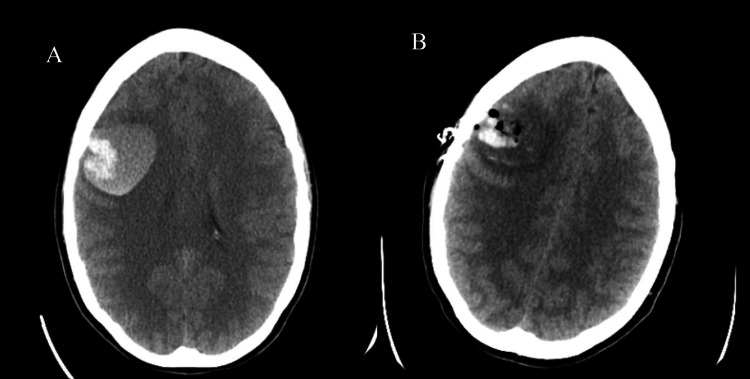
(A) Initial brain CT without contrast shows parenchymal hemorrhage in the right frontal lobe. (B) Post-operative brain CT without contrast shows a decrease in the size of hemorrhage and edema, with minimal left midline shift.

The next day, the patient arrived in the emergency department (ED) with an acute onset of speech difficulty, severe right-sided headache, and nausea. The physical examination was remarkable for dysarthria, left-sided facial droop, and decreased left upper extremity muscle strength. A brain CT without contrast was performed, which showed a parenchymal 4.2-cm hematoma in the right frontal lobe (Figure 4A). The neurosurgery service was consulted, and a right-sided craniotomy with evacuation of the hematoma was performed. The post-operative brain CT showed a right frontal craniotomy with an interval decrease in the size of the hematoma, with some residual blood in the area and vasogenic edema (Figure 4B).

Left-sided weakness and facial droop improved with minimal residual deficits during her hospital course. The patient was discharged with an outpatient rehabilitation referral and close neurosurgical follow-up. It is unclear if brain hemorrhage had any relationship with the patient's underlying cardiac anomalies. Neither imaging nor a cerebral angiogram showed any vascular abnormalities in the brain such as an aneurysm or arteriovenous malformation.

**Figure 5:**
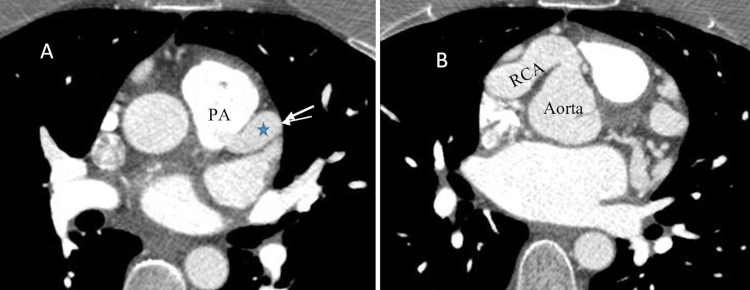
Axial projection of CCTA showing (A) LMCA (blue star) originating from the main pulmonary artery and (B) RCA in normal position and again much dilated. CCTA, coronary computed tomography angiogram; LMCA, left main coronary artery; RCA, right coronary artery

**Figure 6:**
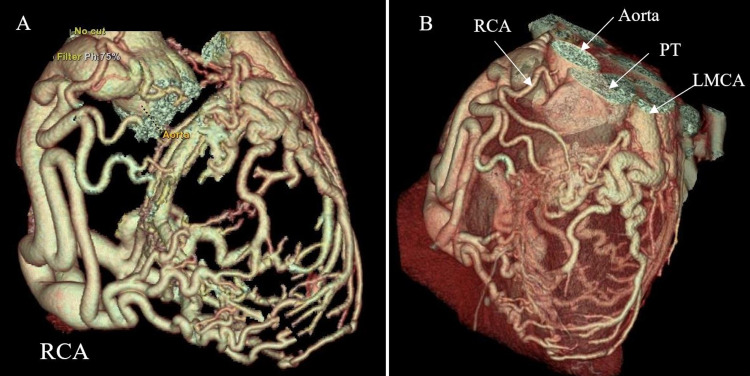
3D volume-rendered CCTA. (A) Dilated and tortuous RCA with multiple collaterals extending to the left ventricle. (B) While the RCA is clearly arising from the aorta, LMCA is arising from the PT. Also, the LMCA is observed in smaller size with very limited extension comparing to the RCA. CCTA, coronary computed tomography angiogram; LMCA, left main coronary artery; PT, pulmonary trunk; RCA, right coronary artery

The patient followed up at the tertiary care facility for further management. CCTA was performed to diagnose the patient’s cardiac anomaly, and imaging revealed the LMCA originating from the main pulmonary artery (PA) (Figure 5A), while the RCA was observed in normal position and very dilated (Figure 5B). Three-dimensional volume-rendered CT demonstrated right coronary arterial dilation with innumerous collaterals extending to the left ventricle and maintaining blood flow (Figure 6A). Also, the LMCA was observed to be very small in size compared to the RCA, with limited extension along the left ventricle (Figure 6B). The diagnosis of ALCAPA syndrome was made with these findings. The patient underwent surgical correction with translocation of the anomalous LMCA to the aorta. She recovered well without any post-operative complications.

## Discussion

ALCAPA syndrome is a rare disease involving the anomalous origin of the LCA from the PA, and its presentation in adulthood is even rarer. The connection of the LCA with the main PA and the resultant steal phenomenon are the main diagnostic features of ALCAPA syndrome [3]. The disease can be classified into two types, the infant type and the adult type, each of which has different manifestations and outcomes. In the infant type, there is no or very limited collateral development from the RCA to the LCA, which leads to early signs and symptoms of heart failure and eventually results in serious outcomes including sudden cardiac death in 90% of the cases [3]. As understood with the disease process, the few that survive into adulthood do so by the formation of ample collateral vessels between the LCA and the RCA. Symptom manifestation occurs secondary to the steal phenomenon, as high-pressure blood from the LCA is shunted into the PA, reducing blood supply to the left heart. This eventually progresses to chronic myocardial ischemia with possible development of ventricular arrhythmias and the sequelae of sudden cardiac death. Adult-type ALCAPA seems to present with a wide variety of symptoms including dilated cardiomyopathy (DCM), MR, chest pain, and dyspnea on exertion, as well as acute coronary syndrome and ventricular arrhythmia [4]. There are few cases reported with a diagnosis of ALCAPA in an asymptomatic patient, but it seems to be present in geriatric age groups as well [4]. This might be due to the development of a better collateral circulation with age and adaption of the coronary structure with time. Our case is of a 38-year-old female marathon runner who presented with symptoms of angina and palpitations and was found to have an NSTEMI and atrial fibrillation with RVR. Our patient subsequently developed a hemorrhagic stroke two days after presentation, the cause of which remains unclear.

From what is known about ALCAPA syndrome, it is a diagnosis typically made during infancy, with an estimated incidence of around one in 300,000 live births [5]. The median survival of adult patients without surgical treatment is 35 years of age, with 80% to 90% dying from sudden cardiac death [6]. Our patient is a marathon runner whose medical history consisted only of mitral insufficiency, which was diagnosed at the age of 24 years. The patient denied any history of rheumatic disease in childhood or symptoms suggestive of Kawasaki disease, which was an initial differential diagnosis in the setting of dilated RCA found on imaging. EKG upon initial presentation to the ED was negative for ischemic changes, though this could have been masked by A fib with an RVR. An echocardiogram was also negative for wall motion abnormalities or DCM. Though our patient did not have DCM and never received adequate workup for the cause of her mitral valve insufficiency, data suggest that ALCAPA syndrome should be considered in the differential diagnosis of a patient with the combination of DCM and MR [7].

For suspected ALCAPA syndrome, there are two main modalities used for diagnosis: transthoracic echocardiography (TTE) and CCTA. Based on a small retrospective study involving 11 patients with ALCAPA confirmed by surgery, there was no statistical difference between TTE and CTA for diagnosing ALCAPA (p > 0.05) [8]. The confirmation of ALCAPA syndrome requires three caveats for diagnosis with imaging modalities:

1. dilated and tortuous RCA,

2. visualization of dilated coronary collateral arteries within the interventricular septum or along the surface of the heart, and

3. visualization of the LCA origin from the posterior aspect of the main PA [9].

The treatment of choice for ALCAPA syndrome is surgical repair to improve myocardial perfusion. Varying surgical approaches may be employed and can be classified into two groups: coronary system and coronary system repairs [10]. The preferred surgical option is to restore a two-coronary-artery system in order to correct the coronary steal phenomenon [3]. Data have suggested that conservative management with medical therapy in elderly, asymptomatic patients older than 50 years of age might be a reasonable alternative, as the risk of sudden cardiac death declines with age and the surgical risk increases [4].

## Conclusions

This case brings to light a rare finding, ALCAPA syndrome in an adult, previously diagnosed with MR. Survival into adulthood is made possible by the development of an adequate collateral network of vessels connecting the LCA to the RCA. Even then, overall survival into adulthood is generally cut short due to acute complications from years of insufficient perfusion to the workhorse of the heart, the left ventricle. Once confirmed on imaging, surgical correction is the standard of treatment for adults younger than 50 years, who are largely asymptomatic. In conclusion, mitral valve abnormality in a patient with no significant medical history should raise the flag for further investigation.
